# Bilateral primary breast diffuse large B-cell lymphoma detected by mammography but partially occult on FDG PET/CT: A case report

**DOI:** 10.1016/j.radcr.2026.03.066

**Published:** 2026-05-11

**Authors:** Wejdan Hashim AlMusallam, Abedallatif Alsharif, Suzan farouk Ibrahim Salib, Ahmad Alwabari, Mustafa Alsultan

**Affiliations:** a Radiology Department, AlMoosa Health Group, Ahsaa, Saudi Arabia; b Department of Radiology and Nuclear Medicine, School of Medicine, University of Jordan, Amman, Jordan; c Radiology Department Faculty of Medicine, Ain Shams University, Cairo, Egypt; d Oncology Center, AlMoosa Health Group, Ahsaa, Saudi Arabia

**Keywords:** Bilateral, Breast, Lymphoma, PET

## Abstract

A 52-year-old female presented with complaints of a painful left breast mass. On examination, a tender mass was noted in the left breast. Bilateral diagnostic mammography revealed an irregular, dense mass with indistinct borders in the left breast. A similar but smaller dense mass was also identified in the right breast. Ultrasound examination confirmed a heterogeneous, hypoechoic, irregular, microlobulated, antiparallel mass in the left breast. A smaller mass with similar characteristics was also observed in the right breast. Histopathology of both breast masses revealed bilateral diffuse large B-cell lymphoma. PET/CT demonstrated abnormally increased metabolic activity only in the larger left breast mass, while the contralateral mass was not metabolically avid.

The patient received 6 cycles of R-CHOP chemotherapy. A follow-up mammography study performed 1 year after the initial diagnosis showed complete resolution of the breast masses bilaterally across all imaging modalities.

## Introduction

Lymphoma is defined as a group of malignant neoplasms of lymphocytes. The World Health Organization classification system categorizes lymphomas according to the cell of origin into B-cell, T-cell, or natural killer (NK) cell types. Furthermore, they are defined based on their morphology, immunophenotype, genetic, molecular, and clinical features [1].

Lymphomas are mainly classified into 2 main categories: non-Hodgkin lymphoma (NHL) and Hodgkin lymphoma (HL). NHL is the most common type of lymphoma and accounts for approximately 85% of all lymphomas [2]. Lymphoma can involve lymphatic tissue, bone marrow, or extranodal sites.

One of the extremely rare sites of lymphoma involvement is the breast, due to the relatively low proportion of lymphoid tissue within it [3,4]. Breast lymphoma is classified as either primary or secondary. Primary breast lymphoma occurs in the breast in the absence of a previously diagnosed extramammary lymphoma and without evidence of concurrent widespread disease. In contrast, secondary breast lymphoma represents a manifestation of systemic lymphoma, in which the disease spreads to the breast and often involves multiple lymph node groups and other organs rather than originating in the breast itself [5].

Primary breast lymphoma accounts for less than 1% of all breast neoplasms [6]. Furthermore, it represents less than 0.7% of all non-Hodgkin lymphomas and less than 2.2% of all extranodal non-Hodgkin lymphomas [7,8].

Primary breast lymphoma is classified in the literature into 2 main groups. The first group is usually unilateral and typically affects older women between 60 and 65 years of age. The second group is even rarer and presents with bilateral involvement in younger women [[9], [10], [11]].

Here, we present a case of a 52-year-old woman with histopathologically confirmed bilateral primary breast lymphoma.

## Case

This is a 52-year-old female who presented to the surgery clinic with a complaint of a painful left breast mass for 5 days. The patient denied any prior history of breast lumps, other swellings, fever, trauma, previous breast imaging, biopsy, or breast surgery. There was no family history of breast cancer.

On examination, a tender 2 × 2 cm mass was palpated at the 11 o’clock position of the left breast. There were no associated skin or nipple changes and no nipple discharge.

**Fig. 1:**
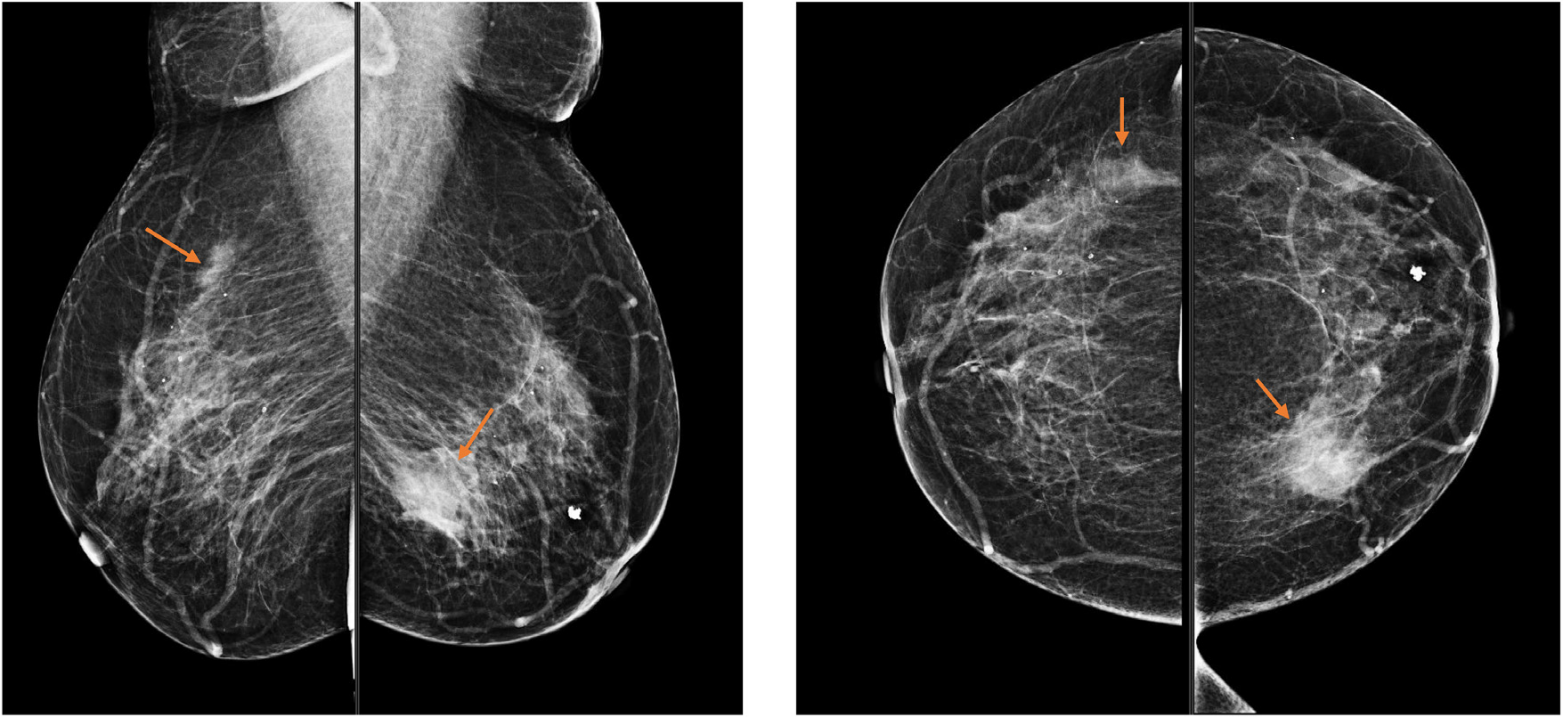
Bilateral breast mammography at the initial presentation demonstrated an irregular dense mass with indistinct borders in the lower inner quadrant of the left breast. A similar but smaller dense mass was also identified in the upper outer quadrant of the right breast.

**Fig. 2:**
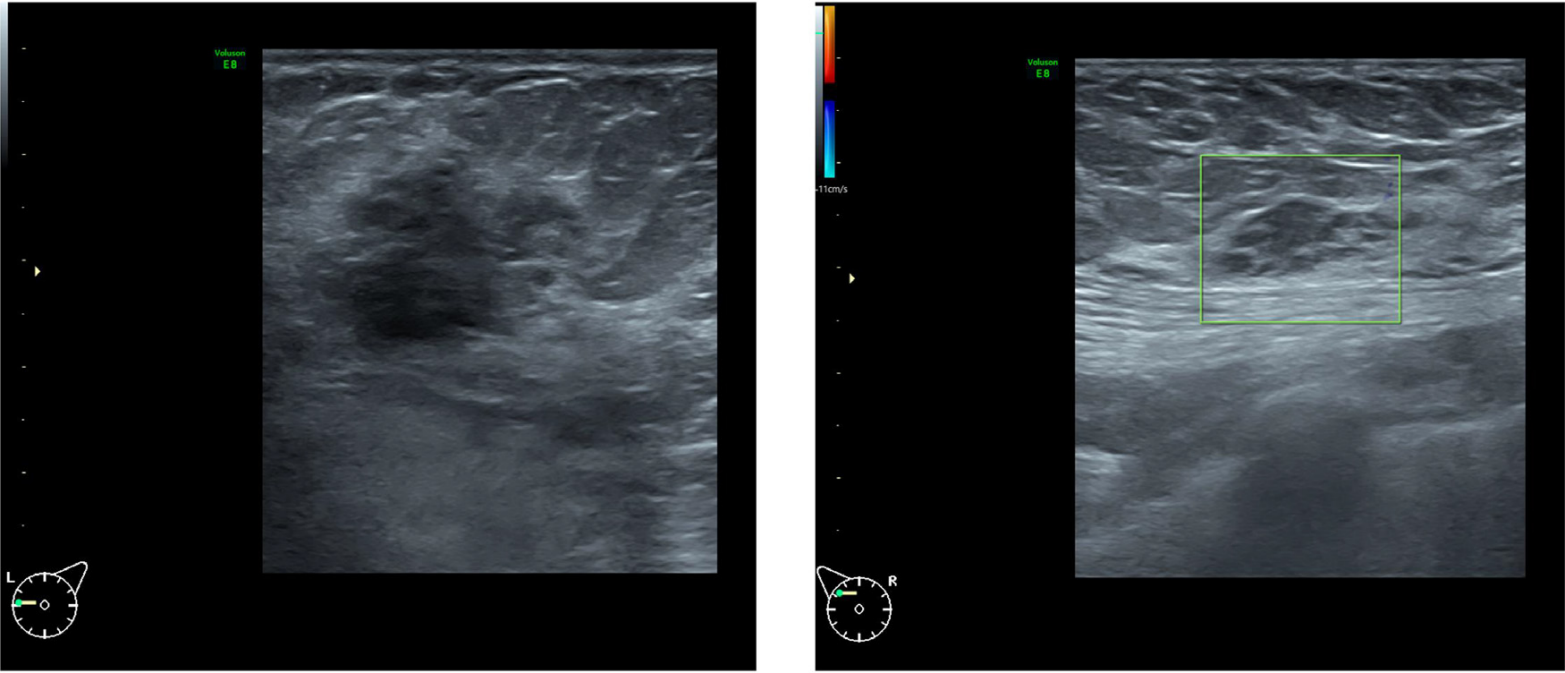
Ultrasound examination demonstrated a heterogeneous, hypoechoic, irregular, microlobulated, antiparallel mass in the left breast ultrasound examination also demonstrated a similar, smaller mass in the right breast.

Bilateral diagnostic mammography and breast ultrasound were performed. Mammography demonstrated scattered fibroglandular densities (ACR B). The left breast showed an irregular, dense mass with indistinct borders. A similar but smaller dense mass was identified in the upper outer quadrant of the right breast (Fig. 1). These findings were confirmed on ultrasound, which revealed a heterogeneous, hypoechoic, irregular, microlobulated mass (Fig. 2).

The findings were categorized as BI-RADS 4; therefore, bilateral breast biopsies were performed. Histopathological examination of the left breast revealed atypical dense lymphoid infiltrates. The right breast biopsy demonstrated benign mammary tissue with focal moderate usual ductal hyperplasia and 1 microcalcification. Immunoglobulin gene rearrangement analysis was performed to exclude a neoplastic lymphoproliferative disorder. B-cell rearrangement was negative for a clonal B-cell neoplasm. Accordingly, the clinical decision was to schedule a follow-up mammogram after 6 months or earlier if clinical progression occurred.

**Fig. 3:**
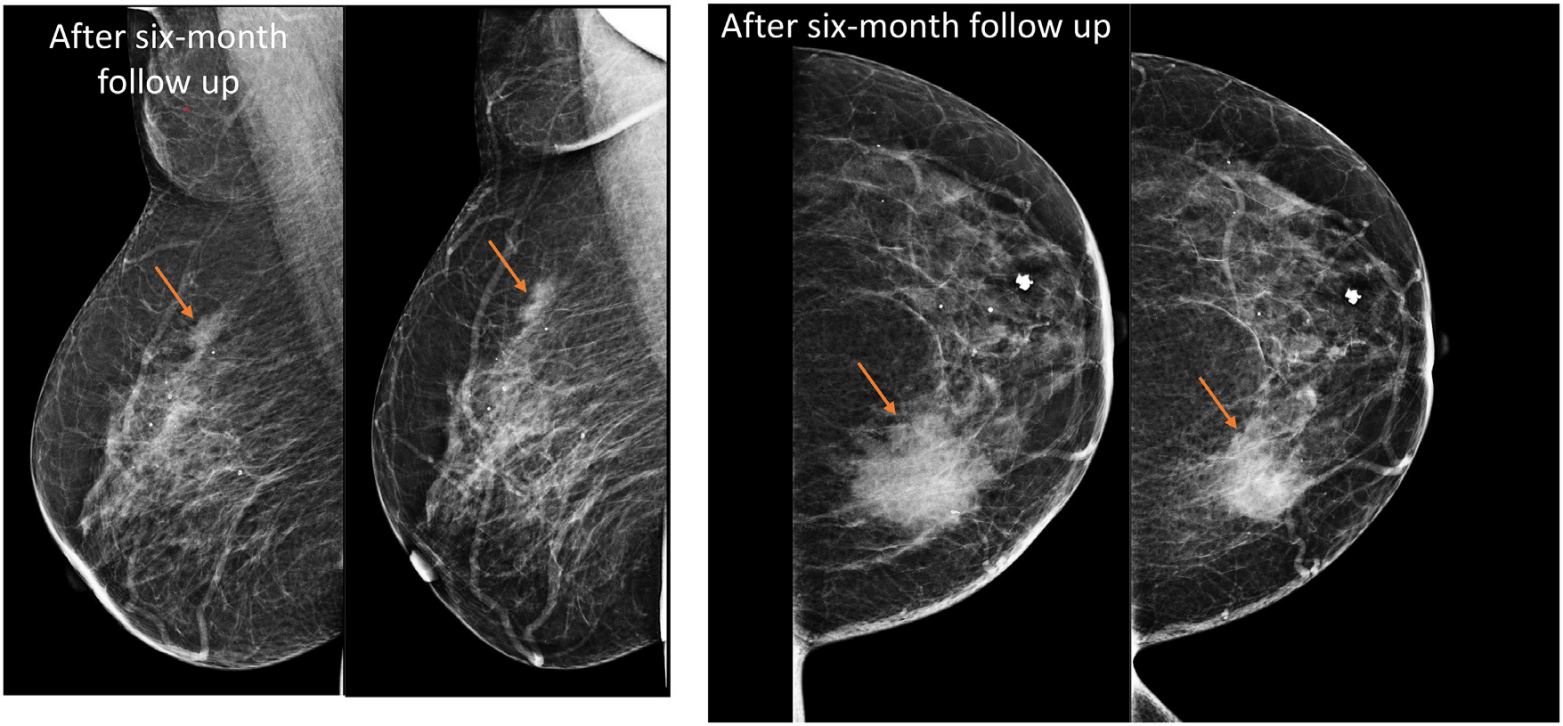
A six-month follow-up mammography study demonstrated an increase in the size of the previously noted masses in both the left and right breasts.

**Fig. 4:**
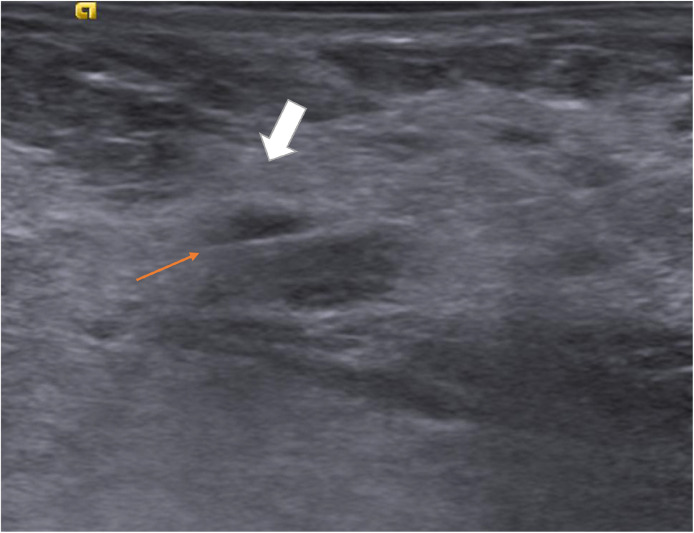
Ultrasound images obtained at the time of biopsy confirmed adequate sampling of the small right breast mass.

A follow-up mammogram at 6 months demonstrated an increase in the size of the previously noted masses in both the left and right breasts (Fig. 3); therefore, repeat biopsies were performed bilaterally (Fig. 4). Histopathological examination confirmed bilateral diffuse large B-cell lymphoma.

**Fig. 5:**
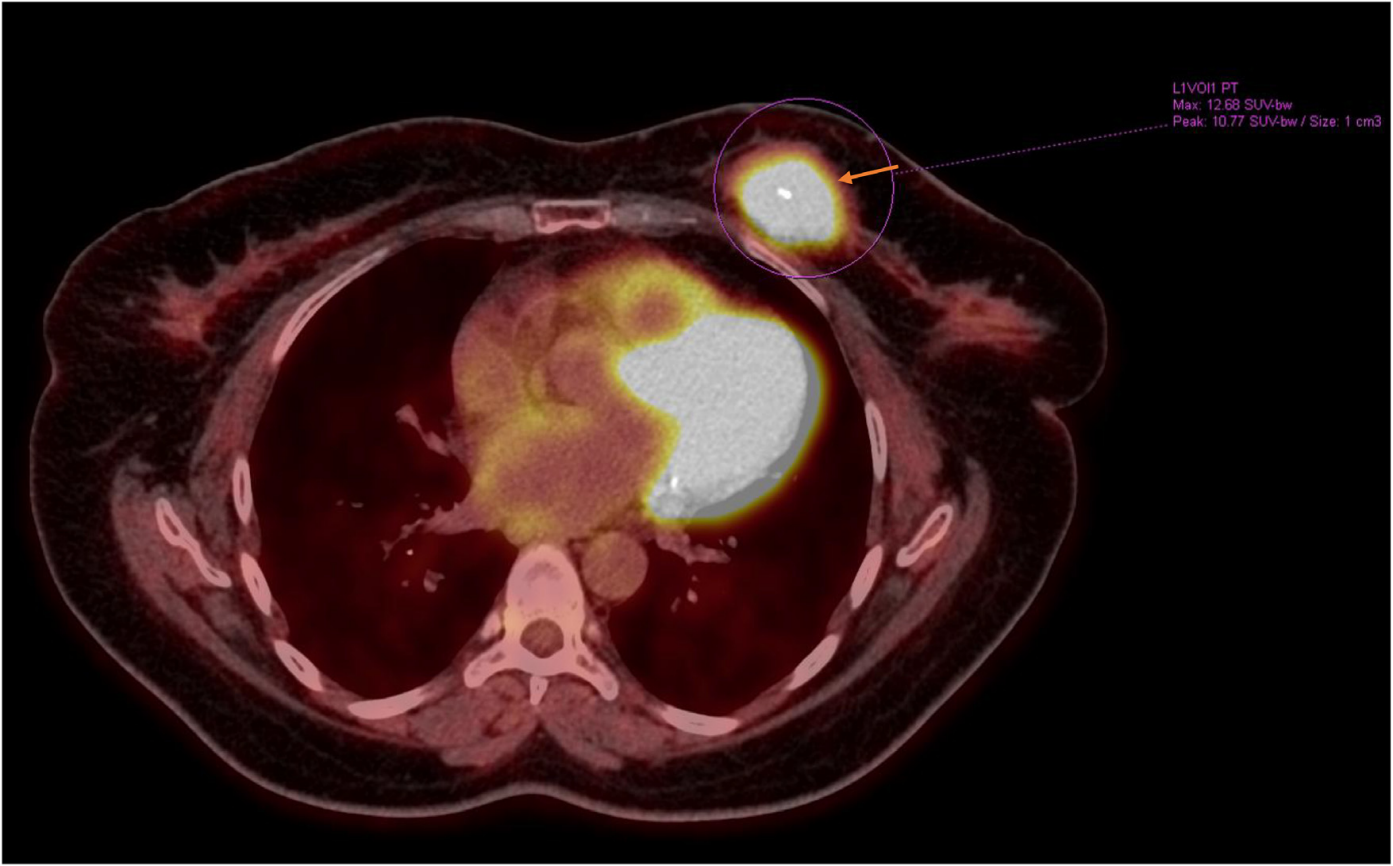
Whole-body 18F-FDG PET scan demonstrated a hypermetabolic, irregular soft tissue mass in the upper outer quadrant of the left breast, with a biopsy marker in place (arrow).

**Fig. 6:**
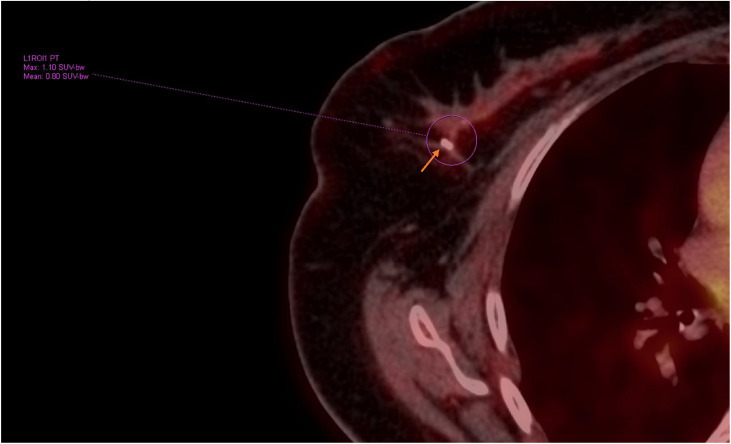
Pre-treatment whole-body 18F-FDG PET scan demonstrated no significant hypermetabolic activity in the right breast SUVmax 1.10, despite histopathologically confirmed diffuse large B-cell lymphoma (arrow indicates the site of the right breast marker).

Further evaluation and staging with a whole-body 18F-FDG PET/CT scan demonstrated a hypermetabolic irregular soft tissue mass in the upper outer quadrant of the left breast, with a biopsy marker in situ (SUVmax 12.7) (Fig. 5). In contrast, there was no hypermetabolic activity in the right breast lesion (SUVmax 1.10), despite the histopathological diagnosis of diffuse large B-cell lymphoma and a measured size of 2.2 × 1.4 cm on ultrasound and mammography (Fig. 6). There was no evidence of additional hypermetabolic breast lesions bilaterally and no hypermetabolic supra- or infradiaphragmatic lymphadenopathy.

**Fig. 7:**
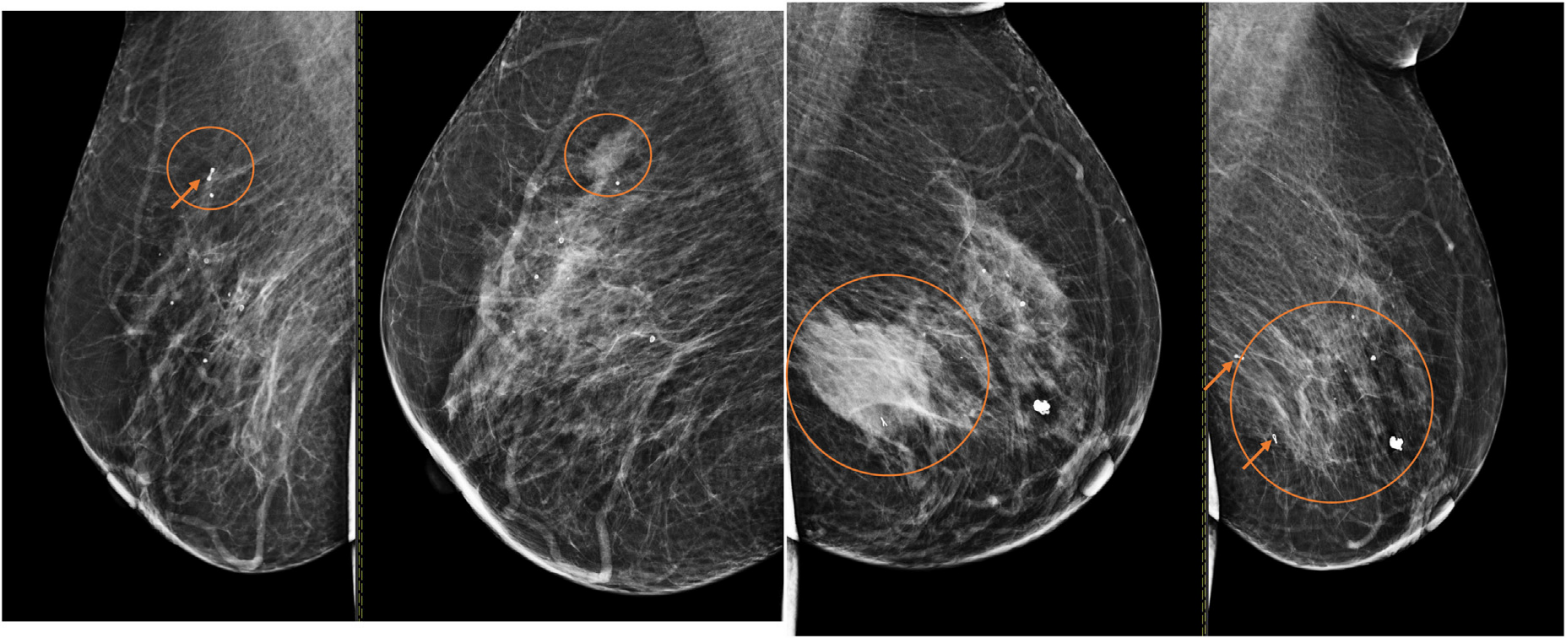
Follow-up mammography demonstrated complete resolution of the masses in the right upper outer quadrant and left lower inner quadrant, with arrows indicating the positions of the post-treatment markers.

**Fig. 8:**
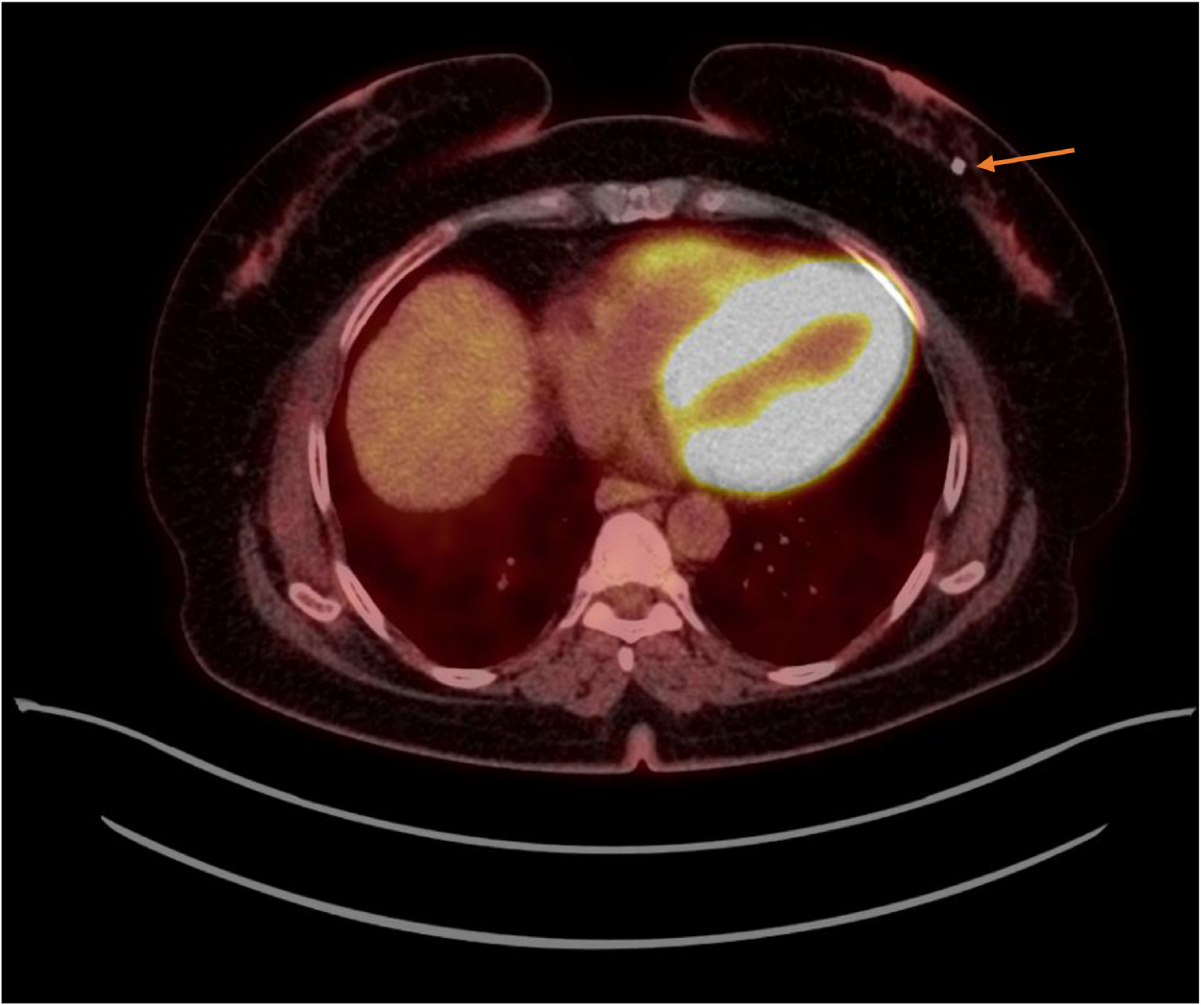
Follow-up whole-body 18F-FDG PET scan showed no evidence of hypermetabolic nodal or breast lymphomatous disease recurrence, with the arrow indicating the left breast marker.

The patient received 6 cycles of R-CHOP (rituximab, cyclophosphamide, doxorubicin, vincristine, and prednisone). Follow-up mammography and 18F-FDG PET/CT performed 1 year after the initial diagnosis demonstrated complete resolution of both breast masses, with biopsy markers remaining in situ (Fig. 7, Fig. 8).

## Discussion

Primary breast lymphoma is generally classified into 2 main groups in the literature. The first, more common group typically presents as a unilateral lesion and predominantly affects older women, usually between 60 and 65 years of age. The second group is much rarer and is characterized by bilateral breast involvement, often occurring in relatively younger women. These classifications help guide clinical suspicion, diagnostic evaluation, and management strategies, given the differences in presentation, prognosis, and treatment response between unilateral and bilateral cases [[9], [10], [11]].

Patients diagnosed with primary breast lymphoma most commonly present with a palpable, painless breast mass, often localized to the lateral quadrants of the breast. Unlike typical presentations of primary breast carcinoma, clinical signs frequently associated with malignant breast tumors—such as skin dimpling or retraction, nipple inversion or discharge, erythema, and ulceration—are relatively uncommon in breast lymphoma. Systemic “B-symptoms,” including fever, night sweats, and weight loss, are also usually absent at presentation, particularly in cases confined to the breast [12,13].

Primary breast lymphoma lacks pathognomonic imaging features across conventional and advanced modalities, including mammography, ultrasound, and magnetic resonance imaging (MRI). On mammography, these lesions typically present as solitary, well-circumscribed or occasionally irregular masses, most often without associated microcalcifications or spiculated margins. Unlike invasive breast carcinoma, architectural distortion and skin or nipple changes are uncommon [14].

Ultrasound evaluation of primary breast lymphoma also demonstrates non-specific imaging features, limiting its diagnostic specificity. A study reviewing 32 patients with breast lymphoma categorized masses into nodular and diffuse subtypes. In this cohort, nodular breast lymphoma typically appeared as well-circumscribed, hypoechoic, and relatively denser masses, whereas diffuse breast lymphoma often presented with ill-defined, heterogeneous, and less compact parenchymal involvement. These findings suggest that while ultrasound can help identify suspicious lesions, it cannot reliably differentiate lymphoma from other breast malignancies [15].

Magnetic resonance imaging (MRI) can serve as a valuable adjunct in the evaluation of breast lymphoma, particularly when findings from mammography and ultrasound are inconclusive. Breast lymphoma on MRI typically appears as an irregular or oval mass with a homogeneous or heterogeneous intermediate T1-weighted signal intensity, high T2 signal intensity, and heterogeneous or rapid contrast enhancement. Diffusion-weighted imaging (DWI) may demonstrate restricted diffusion due to the high cellularity of lymphomatous tissue. MRI is also useful for assessing the extent of disease, multifocality, and involvement of adjacent structures. However, MRI findings are not specific, and histopathological examination remains essential for definitive diagnosis [16,17].

Positron emission tomography (PET) using fluorine-18-deoxyglucose (FDG) has emerged as a valuable non-invasive imaging modality for the diagnosis, staging, and follow-up of lymphoma. FDG-PET demonstrates high sensitivity and specificity in patients with Hodgkin lymphoma (HL) as well as in most subtypes of both indolent and aggressive non-Hodgkin lymphoma (NHL) [18]. In particular, FDG-PET/CT shows excellent sensitivity for diffuse large B-cell lymphoma, with reported sensitivities ranging from 85% to 95% and specificity approaching 95%-100% [19].

FDG PET/CT is highly sensitive for most aggressive lymphomas; however, false-negative results can occur in primary breast lymphoma. Several factors may contribute to lack of FDG avidity, including small mass size, low tumor cellularity, or low metabolic activity of the neoplastic cells. Masses with predominantly fibrotic stroma or minimal proliferative activity may fail to accumulate sufficient FDG to be detected. Additionally, partial-volume effects in small or thin masses can reduce apparent uptake on PET imaging. This case highlights that a metabolically “occult” mass on FDG PET/CT does not exclude malignancy, reinforcing the necessity of combining PET findings with conventional imaging modalities [[20], [21], [22]].

To our knowledge, this is the first reported case of pathologically confirmed primary breast diffuse large B-cell lymphoma (specifically the right breast lesion) that was clearly identified on mammography and ultrasound but was not FDG-avid on PET/CT. This underscores the continued importance of conventional imaging, as mammography and ultrasound remain essential for evaluating both breasts in patients with lymphoma, since bilateral involvement cannot be reliably excluded based solely on FDG-PET/CT findings [23].

## Conclusion

Primary bilateral breast diffuse large B-cell lymphoma is a rare entity with nonspecific clinical and imaging features on mammography and ultrasound. Although FDG PET/CT is generally a highly sensitive modality for the detection and staging of lymphoma, this case demonstrates that some histopathologically proven masses may show little or no metabolic activity. Mammography and ultrasound remain important tools for mass detection, while histopathological examination remains the gold standard for definitive diagnosis, classification, and guidance of appropriate therapy.

## Patient consent

Informed written consent taken from the patient for publication of the case report.
